# A Mini Axial and a Permanent Maglev Radial Heart Pump§

**DOI:** 10.2174/1874120700701010001

**Published:** 2007-05-31

**Authors:** Kun-Xi Qian, Wei-Min Ru, Hao Wang, Teng Jing

**Affiliations:** Inst. BME, Jiangsu Univ. Zhenjiang, 212013, China

## Abstract

The implantability and durability have been for decades the focus of artificial heart R&D. A mini axial and a maglev radial pump have been developed to meet with such requirements.

The mini axial pump weighing 27g (incl.5g rotor) has an outer diameter of 21mm and a length of 10mm in its largest point, but can produce a maximal blood flow of 6l/min with 50mmHg pressure increase. Therefore, it is suitable for the patients of 40-60kg body weight. For other patients of 60-80kg or 80-100kg body weight, the mini axial pumps of 23mm and 25mm outer diameter had been developed before, these devices were acknowledged to be the world smallest LVADs by Guinness World Record Center in 2004.

The permanent maglev radial pump weighing 150g is a shaft-less centrifugal pump with permanent magnetic bearings developed by the author. It needs no second coil for suspension of the rotor except the motor coil, different from all other maglev pumps developed in USA, Japan, European, etc. Thus no detecting and controlling systems as well as no additional power supply for maglev are necessary. The pump can produce a blood flow up to as large as 10l/min against 100mmHg pressure.

An implantable and durable blood pump will be a viable alternative to natural donor heart for transplantation.

## INTRODUCTION

**Fig. (1):**
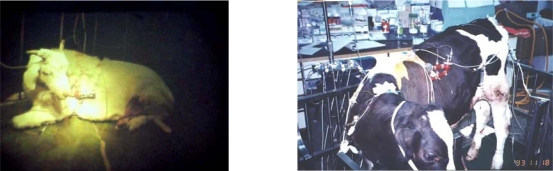
The author’s implantable impeller pump was used as a LVAD in a free-walking artificial heart experimental goat in 1984 (left) and the survival was lasted to over two months on a calf in 1994 with the author’s pump weighing 110g (right).

The R&D of artificial heart has just 50-year history. In the first 25 years from first animal experiment in 1957 to the first human trial in 1982, the work was done to show the feasibility of a man-made mechanical pump, mainly the diaphragm pump, in supporting or replacing the heart function partly or totally. The negative results of the first clinical trial, such as thrombosis in the pump, low quality of patient life because of the bulky driver of the heart pump, forced the people to search the new pumping principle instead of imitating the natural heart. In the second 25 years until now, most attention was attracted to simple and small rotary pumps. The author published the world-first implantable rotary pump in 1983[1]. Then a 240g impeller pump was used in goat in 1984 (Fig. **1**, left), this was the first freely walking artificial heart experimental animal in the history [2]. The survival was lasted to over 2 moths in 1994 during the author’s stay in Taiwan University (Fig. **1**, right) and the weight of the device was reduced to 110g (Chronic left ventricular assist in calves with a pulsatile impeller pump. J American Soc Artif Organ. Vol. 43, No.1, PP89, 1997). Thereafter, many rotary pumps were developed worldwide and a Jarvik 2000 axial pump has worked in a patient for about 7 years [3].

The miniaturization of the heart pump has continuously achieved progress in the author’s laboratory recently. A mini axial pump with 21mm OD and 27g weight has been developed and acknowledged as the world-smallest left ventricular assist device (LVAD) by Guinness World Records Center(Claim ID: 86348, Membership Number: 80362).

The durability has been another key point in improving the heart pump meanwhile. The bearing wear and the heat generation along the bearing, which was recognized to be one of the most possible sites of thrombosis, had limited the rotary heart pump to short-term and/or rescue applications. For eliminating the mechanical contact and wear between the rotor and the stator, other investigators in USA, European, Japan and etc, developed electromagnetic bearings, resulting in need of extra coil and rotor position detection system, feed-back control and additional energy consumption [4-7]. The author insisted in developing permanent magnetic bearing in order to retain the advantages but to avoid the disadvantages of electromagnetic bearings. After the development of some prototypes and experimental models, a permanent maglev radial centrifugal pump was designed, manufactured and tested in the author’s institute.

This paper presents briefly the principle and structure of a mini axial and a maglev radial pump developed by the author in the recent years and calls for cooperation in further R&D of these devices.

## MINI AXIAL PUMP

The mini axial pump is shown in Fig. (**2**). It consists of a stator and a rotor. The stator has a motor coil with iron core and the rotor is composed of a rotor magnets assemble and an impeller. The device weighing 27 gram (incl. rotor 5g) has an outer diameter of 21 mm and a length of 10mm in its largest point, and can be then placed in aortic valve annulus of a patient or an animal with 40-60kg body weight. Thus the pump can deliver the blood directly from the ventricle to aorta without need of inlet and outlet connecting tubs that are thought to be the most favorite sites of thrombus formation. The bench testing with saline demonstrated the pump produced 6 liter per minute flow against 50mmHg pressure increase at its rotating speed of 17,500 rpm. The flow rate and the pressure increase can be adjusted by changing the rotating speed of the pump, *via* increasing or decreasing the input voltage.

The first animal trial in pig(50kg body weight) indicated the device can sewed onto the aortic valve annulus without any harm to adjacent cell, tissue or/and organ function.

For other patients of 60-80kg or 80-100kg body weight, the mini axial pumps of 23mm and 25mm outer diameter had been developed before.

## MAGLEV RADIAL PUMP

**Fig. (3):**
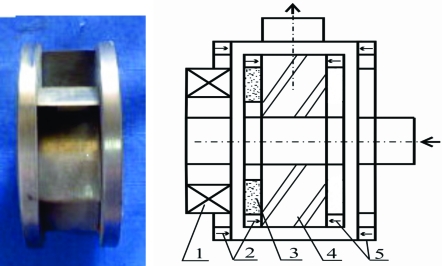
Permanent maglev centrifugal pump (right) and its impeller (left). The left side of the impeller is a magnets disc for rotation and the right side of the impeller is a magnets disc for suspension. The pump weighing 150g has a maximal diameter of 42mm, and its length in largest point is 35mm. This is the smallest maglev centrifugal pump until now according to the author’s knowledge. 1. motor coil; 2. passive magnetic bearing; 3. rotor magnets; 4. impeller; 5. passive magnetic bearing.

To simplify the electrically maglev rotary pumps, a shaft-less full-permanent maglev impeller pump without actively controlled coil for rotor suspension has been developed in author’s Institute (Fig. **3**).

The device consists of a stator and a rotor. The stator has a hard polyurethane housing with cylindrical inner surface; on its left side an axially driven DC motor coil winded on an iron core is connected and on its right side a balancing iron ring is screwed. The rotor is compacted by a magnet disc for rotation (in the left), an impeller (in the middle) and a magnet disc for suspension (in the right). The attractive force between the motor coil iron core and the rotor magnet disc for rotation is balanced by the attractive force between the magnet disk for suspension and the balancing iron ring. Furthermore, two novel patented permanent magnetic bearings are devised on both sides of the rotor, eliminating the remaining attractive forces and preventing the rotor being affiliated axially to the stator either in the left or in the right. Each bearing is composed of a small and a big permanent magnetic ring, the small ring is inlaid into the rotor and the big ring is buried in the stator. Two rings magnetized in the same axial direction reject each other, providing an axial bearing force. The attractive force between the rotor and the stator resists the radial eccentric movement of the rotor and thus serves as a radial bearing. The inlet and outlet of the pump are located respectively in the center of the balancing iron ring and onto the periphery of the PU housing. By the bench testing with water the pump produces a flow as large as 10 l/min with 100mmHg pressure head. The pump weighing 150 g has a maximal diameter of 42 mm and a length of 35 mm (excluding inlet and outlet tubes).

It is concluded that a maglev pump without electromagnet may be possible and is therefore worthy for further more extensive investigation.

## Figures and Tables

**Fig. (2):**
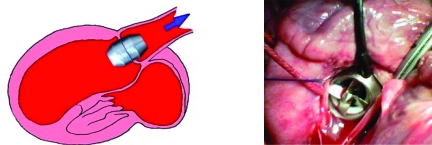
Mini axial pump weighing 27g has an OD of 21mm (left). It delivers blood directly from ventricle to aorta without need of inlet and outlet tubs, which were recognized being the favorite sites of thrombus formation. The first *in vivo* trial demonstrated that the pump can easily sewed onto the aortic valve annulus (right) without harm to adjacent tissue, cell and organ function.
